# CXCL12-CXCR7 axis contributes to the invasive phenotype of pancreatic cancer

**DOI:** 10.18632/oncotarget.11330

**Published:** 2016-08-17

**Authors:** Jun-Chao Guo, Jian Li, Li Zhou, Jian-Yu Yang, Zhi-Gang Zhang, Zhi-Yong Liang, Wei-Xun Zhou, Lei You, Tai-Ping Zhang, Yu-Pei Zhao

**Affiliations:** ^1^ Department of General Surgery, Peking Union Medical College Hospital, Chinese Academy of Medical Sciences/Peking Union Medical College, Beijing 100730, China; ^2^ Department of Pathology, Peking Union Medical College Hospital, Chinese Academy of Medical Sciences/Peking Union Medical College, Beijing 100730, China; ^3^ Department of Pancreatic Cancer, Tianjin Medical University Cancer Institute & Hospital, Tianjin 300060, China; ^4^ Department of Biliary-Pancreatic Surgery, Ren Ji Hospital, School of Medicine, Shanghai Jiao Tong University, Shanghai 200240, China; ^5^ State Key Laboratory of Oncogenes and Related Genes, Shanghai Cancer Institute, Ren Ji Hospital, School of Medicine, Shanghai Jiao Tong University, Shanghai 200240, China

**Keywords:** pancreatic cancer, invasive phenotype, prognosis, CXCL12, CXCR7, mTOR

## Abstract

Chemokine (C-X-C motif) receptor 7 (CXCR7) and its ligand, chemokine (C-X-C motif) ligand 12 (CXCL12), were established to be involved in biological behaviors and associated with prognosis in many cancers. However, effects, underlying mechanisms of CXCL12-CXCR7 axis in invasive phenotype of pancreatic cancer (PC) and its clinicopathologic significances have not been comprehensively explored. In the present study, it was first found by tissue microarray-based immunohistochemistry that CXCL12 and CXCR7 staining scores were significantly associated with vessel invasion and overall survival in two independent cohorts of PC. Besides, co-expression of these proteins was an independent prognosticator in multivariate analysis in both cohorts. Then, migration and invasion, but not proliferation, were decreased in CXCR7-stably silenced PC cells, whereas opposite changes were observed in CXCR7-stably overexpressed cells, accompanied by alterations of mTOR and Rho/ROCK pathways. CXCL12 stimulated migration, invasion, CXCR7 expression and phosphorylation of key mTOR proteins. AMD3100 did not influence effects of CXCL12. Two mTOR inhibitors, rapamycin and Torin1, reversed enhanced invasive phenotypes and mTOR phosphorylation in CXCR7-overexpressed cells. Moreover, CXCR7 directly interacts with mTOR. Finally, liver metastasis, but not growth, was affected by CXCR7 status in orthotopically-implanted PC models in nude mice. Collectively, CXCL12-CXCR7 axis accelerates migration and invasion of PC cells through mTOR and Rho/ROCK pathways, and predicts poor prognosis of PC.

## INTRODUCTION

It has been well known that pancreatic cancer (PC) carries extremely disappointing overall prognosis, despite fully resected early lesions [1]. The high ratio of advanced (metastatic or regional spread) disease at diagnosis (more than 70%) might account, at least in part, for this unsatisfactory long-term outcome [1, 2]. Therefore, molecules and mechanisms relative to invasion and dissemination of PC cells are long of interest. Except for classical signaling pathways involved in pancreatic tumorigenesis, for example, Ras-ERK pathway [3], many genes/proteins, such as NOP14 [4, 5], DCLK1 [5], interleukin-22/interleukin-22 receptor [6], FoxQ1 [7], CHIP [8] and microRNAs [9], were recently found to play important roles in migration and invasion of PC cells. In addition, some of them were shown to be of prognostic value in PC [6, 8, 9]. However, further data concerning exact mechanisms and more candidates remain to be accumulated.

Nowadays, important roles of chemokines, chemoattracting proteins binding to and activating their corresponding receptors, in malignant behaviors of cancer cells have been evident [10]. Structurally, chemokines are classified into four families, i.e., CXC, CC, CX3C, and C [10]. Among them, CXC ligand 12 (CXCL12, also called stromal-derived factor-1), one of CXC chemokines, was previously shown to have important impacts on proliferation and invasion of many types of cancer cells, via its specific receptor, CXCR4 [10, 11]. In fact, CXCR4 was long regarded as the exclusive receptor for CXCL12 [11]. However, another high affinity receptor of CXCL12, CXCR7, has recently been identified [12]. It has been suggested that CXCR7 is involved in a broad range of phenotypes of cancer, such as growth, migration, chemotaxis, adhesion and spreading [11]. In PC, most articles indicated that CXCL12 promotes proliferation, invasion and chemoresistance [13–20], mainly through CXCR4 [14–16, 18–20]. It was also revealed that MAPK, NF-*κ*B, FAK, ERK, Akt, Wnt and non-canonical Hedgehog pathways as well as extracellular matrix degradation enzymes are involved in relative mechanisms [14, 16, 18–20]. The histological observations about expression of CXCL12 in pancreatic intraepithelial neoplasia and PC as well as its prognostic significance in PC support its role as a tumor promoter [21–23]. However, it remains to be controversial, because reverse evidence was also reported [24–26]. On the other hand, CXCR7 was shown to impact cell proliferation in PC [27], but its prognostic value remains controversial [23, 28, 29]. Our previous study identified CXCR7 as a top up-regulated gene in 7,12-dimethylbenzanthraene (DMBA)-induced PC model in rats and preliminarily found its possible impact on migration and invasion of PC cells [30]. Thus far, biological effects, relative mechanisms and clinicopathological significances of CXCL12-CXCR7 axis in PC, especially for invasive proclivity, have not been comprehensively investigated. The present investigation aimed to address the issues, based on histological, in vitro and in vivo experiments.

## RESULTS

### Expression, clinicopathologic and prognostic significances of CXCL12 and CXCR7 in two cohorts of PC

**Figure 1:**
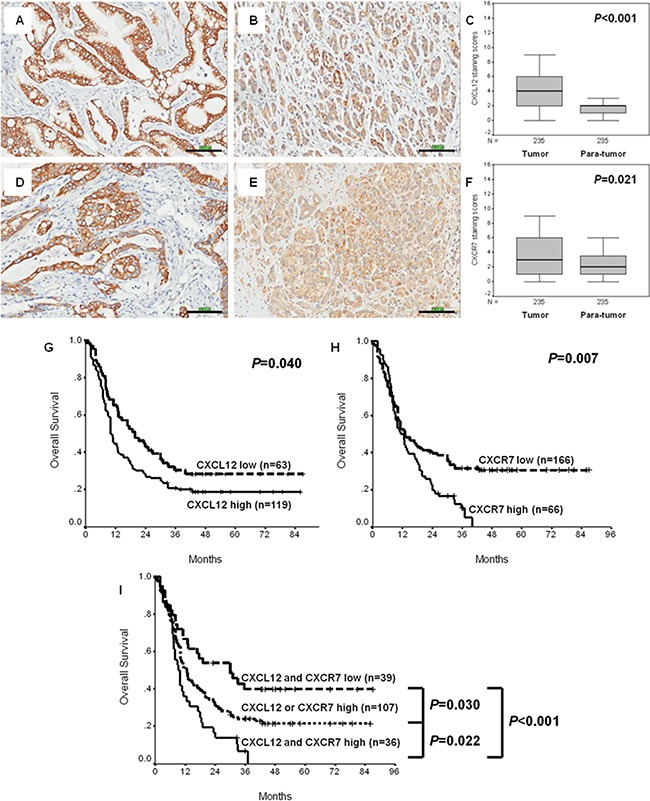
Expression, clinicopathologic and prognostic significances of CXCL12 and CXCR7 in Beijing cohort of PC **A.** High CXCL12 expression in tumor tissues (×200). **B.** High CXCL12 expression in non-tumor tissues (×200). **C.** Staining ranks of CXCL12 in tumor tissues were statistically higher than those in non-tumor ones. **D.** High CXCR7 expression in tumor tissues (×200). **E.** High CXCR7 expression in non-tumor tissues (×200). **F.** Staining ranks of CXCR7 in tumor tissues were statistically higher than those in non-tumor ones. **G.** High CXCL12 expression in tumor tissues predicted poor overall survival. **H.** High CXCR7 expression in tumor tissues predicted poor overall survival. **I.** Combined evaluation of CXCL12 and CXCR7 significantly discriminates favorable, moderate and poor overall survival.

As shown in Figures 1A-1F and Supplementary Figures S1A-S1F, which were derived from Beijing and Shanghai cohorts respectively, staining ranks of CXCL12 and CXCR7 in tumor tissues were statistically higher than those in non-tumor ones (*P*<0.001 and <0.001 for CXCL12; *P*=0.021 and =0.008 for CXCR7; Mann-Whitney *U*-test). Chi-square analysis found that tumoral expressions of CXCL12 and CXCR7 were all significantly associated with vessel invasion in both cohorts′ (*P*=0.007 and =0.047 for CXCL12; *P*=0.022 and =0.003 for CXCR7; Supplementary Tables S1, S2). In Beijing cohort, CXCL12 expression was related to histological grade (*P*=0.019; Supplementary Table S1), whereas CXCR7 expression was linked to sex and histological grade in Shanghai cohort (*P*=0.033 and =0.045; Table S2). By Kaplan-Meier method and log-rank test, high CXCL12 and CXCR7 expressions in tumor tissues predicted poor overall survival in two cohorts (*P*=0.040 and =0.037 for CXCL12; *P*=0.007 and =0.020 for CXCR7; Figures 1G, 1H, Supplementary Figures S1G, S1H, Tables 1 and Supplementary Figure S3). Multivariate Cox regression analysis did not prove the independent impact of CXCL12 or CXCR7 on overall survival in both Beijing and Shanghai cohorts (*P*=0.088 and =0.054 for CXCL12; *P*=0.054 and =0.062 for CXCR7; Tables 1 and Supplementary Figure S3). However, patients with high tumoral CXCL12 and CXCR7 expressions carried poorest prognosis (Figures 1I and Supplementary Figure S1I). Besides, combined high expression of the proteins was identified as an independent prognostic marker (Beijing cohort: HR: 1.456, 95%CI: 1.087-1.950, *P*=0.012; Shanghai cohort: HR: 1.675, 95%CI: 1.108-2.532, *P*=0.014).

**Table 1:** Univariate and multivariate analyses of prognostic factors of PC (Beijing cohort)

Variables	n	Univariate			Multivariate		
		median±SE	95% CI	*P*	HR	95% CI	*P*
Age				0.802			
≥65 years	61	10.0±2.6	5.0-15.0				
<65 years	121	13.0±1.4	10.3-15.7				
Sex				**0.001**			**0.012**
Male	119	11.0±1.1	8.9-13.1		1.705	1.124-2.584	
Female	63	18.9±7.6	4.0-33.8		1		
Tumor location				0.635			
Head	104	13.0±2.6	7.9-18.1				
Non-head	73	12.5±1.7	9.3-15.7				
Tumor size				0.917			
>4cm	70	11.0±1.2	8.6-13.4				
≤4cm	109	13.7±2.9	8.0-19.4				
Histological grade				**0.026**			**0.016**
G1-2	113	15.0±2.5	10.0-20.0		1		
G3-4	53	9.2±1.1	7.1-11.3		1.617	1.092-2.395	
**Vessel invasion**				**0.028**			
Present	82	10.0±0.6	8.7-11.3				
Absent	98	17.4±2.8	11.9-22.9				
T stage				0.452			
T1-2	112	13.0±2.6	7.8-18.2				
T3	68	12.5±2.1	8.5-16.5				
N stage				**0.009**			**0.036**
N0	92	17.4±4.8	8.0-26.8		1		
N1	78	11.0±1.7	7.6-14.4		1.482	1.027-2.140	
Tumoral CXCL12				**0.040**			0.088
High	119	10.0±0.6	8.7-11.3		1.412	0.950-2.098	
Low	63	19.8±4.5	11.0-28.6		1		
Tumoral CXCR7				**0.007**			0.054
High	66	11.2±1.6	8.0-14.4		1.466	0.994-2.162	
Low	116	13.0±2.4	8.3-17.7		1		

NOTE: Partial data are not available, and statistics were based on available data. *P* values were derived from the Log-rank test (univariate) and Cox regression analysis (multivariate).

### CXCR7 promotes migration and invasion of PC cells, involving activation of mTOR signaling pathway

**Figure 2:**
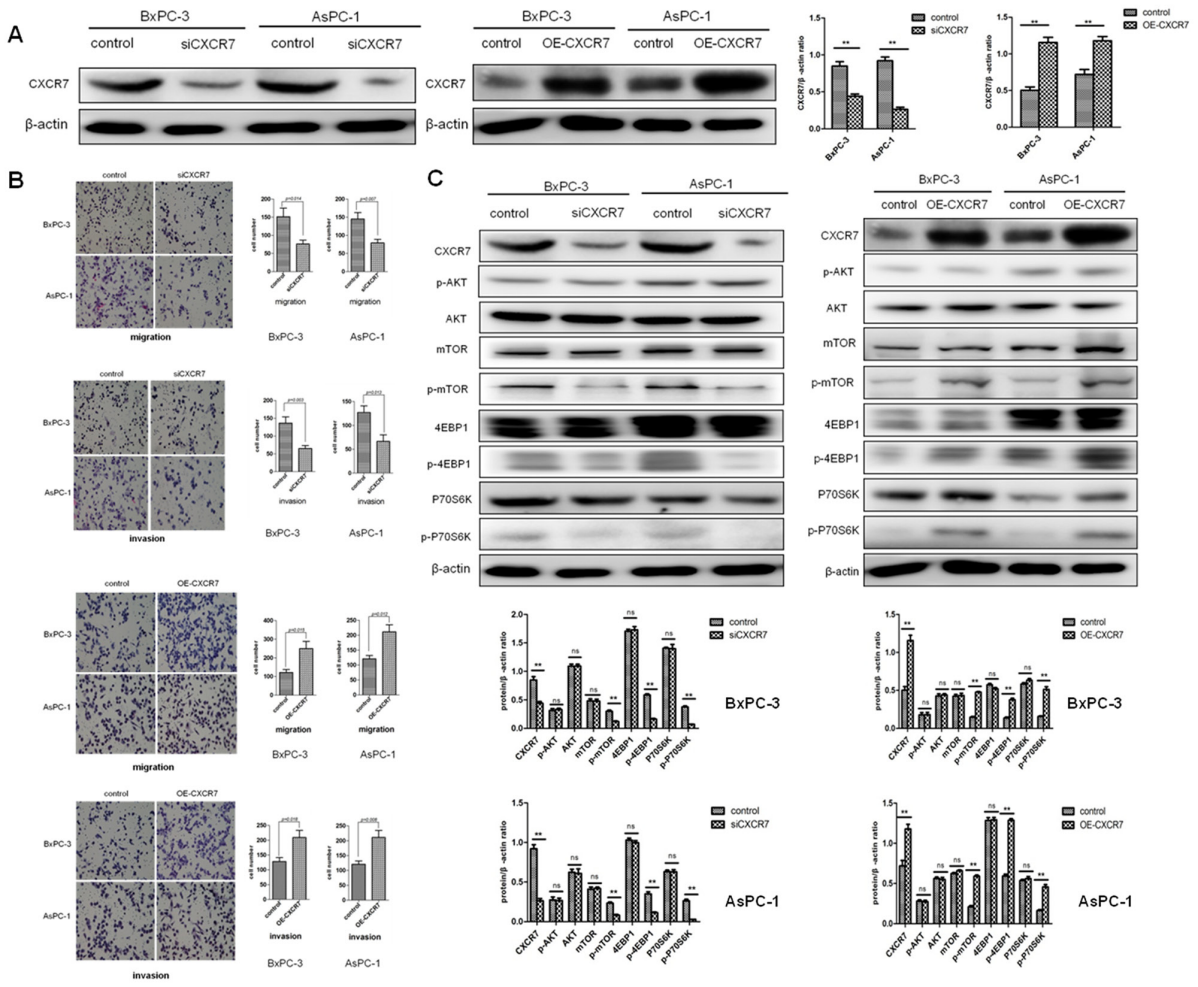
CXCR7 promotes migration and invasion of PC cells, involving activation of mTOR signaling pathway **A.** Successful establishment of CXCR7 stably silenced and overexpressed BxPC-3 and AsPC-1 cells. **B.** Stable silence of CXCR7 decreases cell migration and invasion, while its overexpression has the opposite effects. **C.** Stable silence of CXCR7 inhibits phosphorylation, but not expression of mTOR, 4EBP1 and P70S6K, whereas its overexpression promotes this alteration.

In six tested PC cell lines in that similar baseline expression of CXCR7 was shown (Supplementary Figure S2), BxPC-3 and AsPC-1 were selected to be used in further experiments. Firstly, CXCR7 stably silenced and overexpressed PC sub-lines were successfully established (Figure 2A). Then, it was found that migration and invasion of CXCR7 stably silenced cells were significantly decreased, compared with controls, whereas cells with CXCR7 overexpression had the opposite trend (*P*=0.014 and =0.007 for migration in BxPC-3 and AsPC-1; *P*=0.003 and =0.013 for invasion in BxPC-3 and AsPC-1; Figure 2B). However, no alterations in proliferation of different cell types were observed (All *P*>0.05; Supplementary Figure S3). Western blotting revealed that CXCR7 silencing and overexpression influenced phosphorylation, rather than total protein expression, of three key components of mTOR signaling pathway, i.e. mTOR, 4EBP1 and P70S6K (Figure 2C), but there were not changes in Akt (Figure 2C) and a large panel of CXCR7 associated proteins that were previously reported (Supplementary Figure S4).

### CXCL12 accelerates migration and invasion of PC cells through CXCR7 associated activation of mTOR signaling pathway

**Figure 3:**
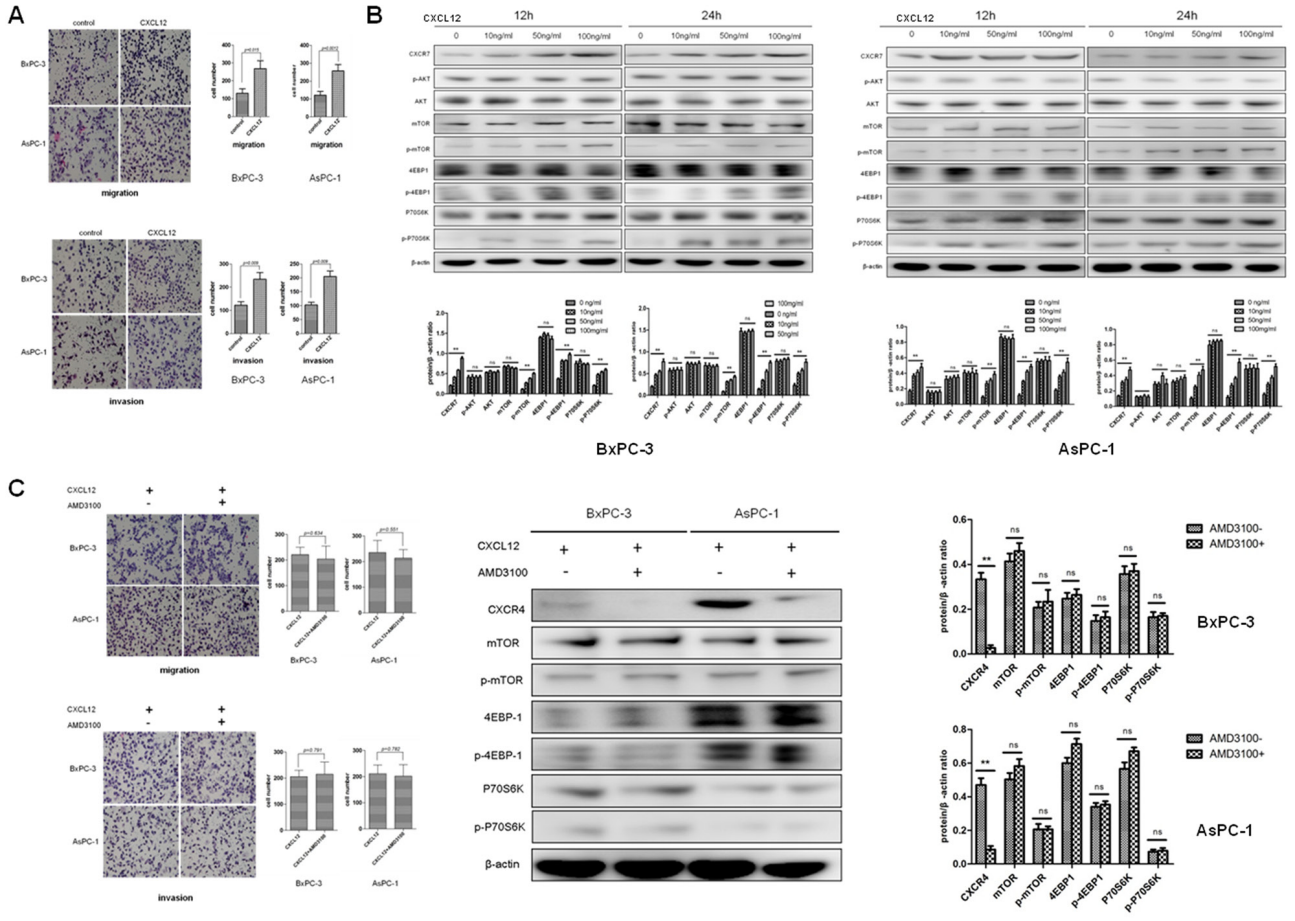
CXCL12 accelerates migration and invasion of PC cells through CXCR7 associated activation of mTOR signaling pathway **A.** CXCL12 enhances migration and invasion of BxPC-3 and AsPC-1 cells. **B.** CXCL12 elevates phosphorylation of mTOR, 4EBP1 and P70S6K in a dose-dependent manner. **C.** In CXCL12 treated cells, AMD3100, a specific CXCR4 inhibitor, does not affect migration, invasion and expression, in particular phosphorylation of mTOR, 4EBP1 and P70S6K, except for CXCR4 expression.

To study the impact of CXCL12 on invasive potential of PC cells, BxPC-3 and AsPC-1 were treated with recombinant CXCL12. It was demonstrated that migration and invasion were significantly enhanced by CXCL12 in both cell lines (*P*=0.015 and =0.0012 for migration in BxPC-3 and AsPC-1; *P*=0.009 and =0.009 for invasion in BxPC-3 and AsPC-1; Figure 3A). Besides, CXCL12 increased CXCR7 expression and phosphorylation of aforementioned three components of mTOR signaling pathway, mTOR, 4EBP1 and P70S6K, but not Akt, basically in a dose-dependent manner (Figure 3B). In CXCL12 treated cells, the use of AMD3100 (1.0ng/mL), a specific inhibitor of the alternative receptor of CXCL12, CXCR4, did not affect migration and invasion as well as phosphorylation of mTOR, 4EBP1 and P70S6K (*P*=0.634 and =0.551 for migration in BxPC-3 and AsPC-1; *P*=0.791 and =0.782 for invasion in BxPC-3 and AsPC-1; Figure 3C).

### Effects of mTOR signaling pathway inhibition on CXCR7-mediated migration and invasion of PC cells and relative molecular mechanisms

**Figure 4:**
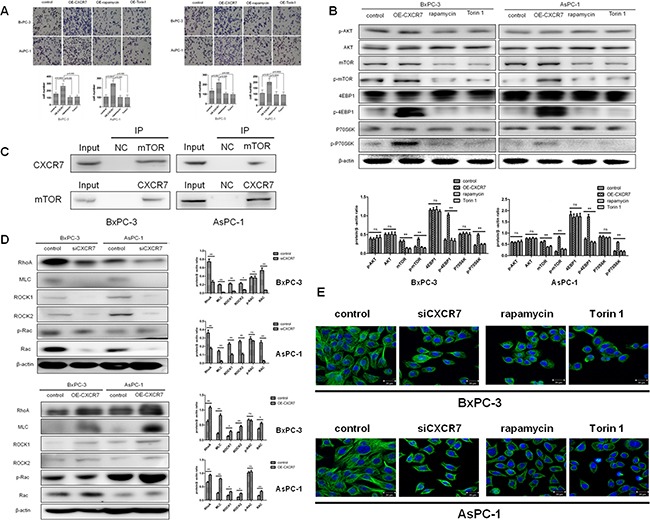
Effects of mTOR signaling pathway inhibition on CXCR7-mediated migration and invasion of PC cells and relative molecular mechanisms **A.** Two mTOR inhibitors, rapamycin and Torin1, reverse CXCR7 induced migration and invasion of PC cells. **B.** Rapamycin and Torin1 reverse phosphorylation of mTOR, 4EBP1 and P70S6K, but do not influence Akt. **C.** Immunoprecipitation showed that CXCR7 directly interacts with mTOR. **D.** Stable silence and overexpression of CXCR7 down- and up-regulate expression of RhoA, MLC, Rac, p-Rac, ROCK1 and 2. **E.** Immunocytochemical staining of F-actin showed that CXCR7 silence, rapamycin and Torin1 disorganize and depolymerize cytoskeleton in PC cells.

**Figure 5:**
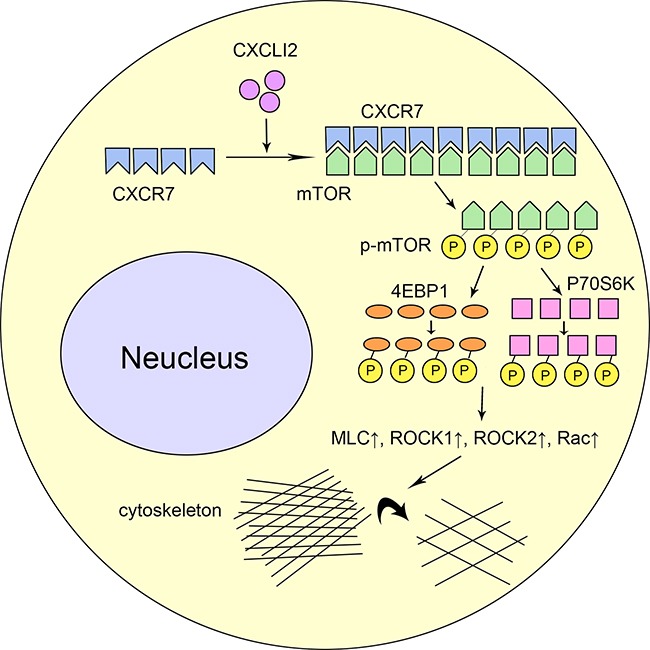
A diagram illustrating the related molecular mechanism of CXCL12-CXCR7 axis in PC

To explore the exact role of mTOR signaling pathway in CXCR7-mediated migration and invasion of PC cells, two mTOR inhibitors, rapamycin and Torin1 were used to treat CXCR7 stably overexpressed cells. It was found that both rapamycin and Torin1 decreased migration and invasion that were significantly increased by CXCR7 overexpression (*P*=0.000, =0.000, =0.000 and =0.000 for rapamycin and Torin1 in migration of BxPC-3 and AsPC-1; *P*=0.000, =0.000, =0.0002 and =0.0003 for rapamycin and Torin1 in invasion of BxPC-3 and AsPC-1; Figure 4A). Then, the two mTOR inhibitors also reverse up-regulated expression of p-mTOR, p-4EBP1 and p-P70S6K, but not Akt and p-Akt, in CXCR7 stably overexpressed cells (Figure 4B). Immunoprecipitation showed that CXCR7 directly interacts with mTOR (Figure 4C). Finally, the down- and up-regulated expression of proteins in Rho/ROCK pathway that was associated with mTOR, including RhoA, MLC, Rac, p-Rac, ROCK1 and 2, was also observed in CXCR7 stably silenced and overexpressed cells (Figure 4D). Based on above in vitro experiments, a diagram illustrating the related molecular mechanism of CXCL12-CXCR7 axis in PC was shown in Figure 5.

### CXCR7 facilitates hepatic metastasis, but not growth, of PC in nude mice

**Figure 6:**
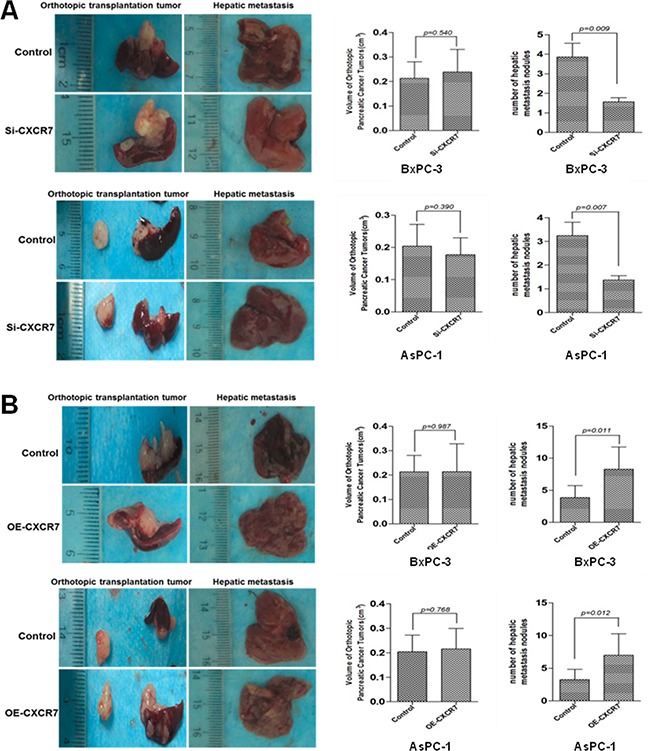
CXCR7 promotes hepatic metastasis, but not orthotopic growth, of PC in nude mice **A.** The hepatic metastatic nodule numbers, but not orthotopic tumor volumes, of CXCR7-silenced cells are significantly less than those of controls. **B.** CXCR7-overexpressed cells generate more hepatic metastatic nodules, rather than faster orthotopic tumor growth, in contrast to controls.

To confirm the roles of CXCR7 in PC in vivo, an orthotopic implantation model was generated, and hepatic metastasis was seen (Supplementary Figure S5). Hepatic metastatic nodule number, but not primary tumor growth (All *P*>0.05; Figure 6A), of CXCR7-silenced cells was significantly less than that of controls (*P*=0.009 and =0.007 in BxPC-3 and AsPC-1; Figure 6A). On the other hand, CXCR7-overexpressed cells made more hepatic metastatic nodules, rather than faster primary tumor growth (All *P*>0.05; Figure 6B), in contrast to controls (*P*=0.011 and =0.012 in BxPC-3 and AsPC-1; Figure 6B).

## DISCUSSION

It has been demonstrated that CXCL12 and CXCR7, another high affinity receptor of CXCL12 [12], except for CXCR4, play pivotal roles in growth, migration, chemotaxis, adhesion and spreading of cancers [11]. Although CXCL12 and CXCR7 were revealed to be associated with outcomes in some kinds of cancer [31, 32], data about their prognostic values in PC, one of most lethal malignancies [1], remain to be exploratory, even controversial. Thus far, CXCL12 was only found to be prognostic in stage II patients [22], while CXCR7 was surprisingly linked to higher grading but earlier T stage [29], and different impacts on prognosis, especially in combination with CXCR4 [28, 29]. More importantly, CXCL12 and CXCR7 have not been comprehensively evaluated. In the present study, immunohistochemical staining of CXCL12 and CXCR7 in two independent cohorts all discovered their higher expressions in tumor than non-tumor tissues (Figures 1A-1F and Supplementary Figure S1A-S1F). Coupled with the associations between the two proteins and vessel invasion, these histological findings support their oncogenic roles in PC, especially for invasive power. As for the relationships between CXCL12 or CXCR7 and other parameters in a single cohort, they need to be further validated. Furthermore, CXCL12 and CXCR7 were also prognostic (Figures 1G, 1H, Supplementary Figure S1G, S1H, Tables 1 and Supplementary Figure S3). The results are consistent with that for CXCL12 [22], but strengthen prognostic implication of CXCR7 in PC, compared with previous articles [28, 29]. What calls for special attention is that CXCL12 or CXCR7 lost impact on patient survival in multivariate Cox regression test (Table 1 and Supplementary Figure S1). However, concomitant high expression of these proteins carried poorest survival (Figures 1I and Supplementary Figure S1I), and possessed independent prognostic significance in both cohorts. These results, especially the verification between independent cohorts, confirm in tissue level the powerful influences of CXCL12-CXCR7 axis on prognosis of PC. Therefore, biological roles of the axis in PC, except for its potential as a novel promising molecular prognostic marker added to those previously summarized [33, 34], are of interest.

In PC, most authors mainly focused on the effects of CXCL12-CXCR4 axis in cell proliferation, invasion and chemoresistance [14–16, 18–20]. Relative signal transduction pathways include MAPK, NF-*κ*B, FAK, Akt, Wnt and non-canonical Hedgehog, etc [14, 16, 18–20]. For the biological functions of CXCL12 and CXCR7 in PC, existing reports have concerned their boosting impacts on cell proliferation [27]. However, Roy and colleagues found that CXCL12 serves as a tumor suppressor in PC [26]. Therefore, this topic might be controversial. Our data derived from a rat DMBA-induced PC model and human PC cell lines supported the positive role of CXCR7 in cell migration and invasion [30]. To explore the effects of CXCL12-CXCR7 axis and exact mechanisms in PC, we first established sub-lines in that CXCR7 was stably silenced and overexpressed (Figure 2A), based on two PC cell lines, BxPC-3 and AsPC-1. Further Transwell experiments revealed that stable silence of CXCR7 significantly impairs migration and invasion, whereas CXCR7 overexpression promotes the phenotypes (Figure 2B). On the other hand, cell proliferation was not altered (Supplementary Figure S3). These results provide systemic evidence, in contrast to the preliminary clue [30], for the impact of CXCR7 on invasive potential, rather than growth, of PC. Surprisingly, the authors failed to discover changes in Akt (Figure 2C) and a large pool of proteins that were reported to be associated with CXCR7 in cancer (Supplementary Figure S4), but showed those in phosphorylation of mTOR, 4EBP1 and P70S6K (Figure 2C), indicating that mTOR activation might be involved in CXCR7-mediated migration and invasion in PC.

Then, significantly increased cell migration and invasion were observed, accompanied by elevated CXCR7 expression and phosphorylation of mTOR, 4EBP1 and P70S6K, but not Akt, after treated with recombinant CXCL12 (Figures 3A-3B). To determine the role of CXCR4, another receptor of CXCL12, AMD3100, a specific CXCR4 inhibitor, was used in CXCL12 treated cells. Consequently, there were not alterations in migration, invasion and phosphorylation of mTOR, 4EBP1, P70S6K, except for down-regulation of CXCR4 (Figure 3C). Thus, it could now be summarized that CXCL12-CXCR7 axis, not CXCL12-CXCR4 one, promotes migration and invasion of PC, through activation of mTOR, but not Akt in its upstream. These results support the definitions of CXCL12 and CXCR7 as onco-proteins. Furthermore, the current study first established a CXCL12-CXCR7-mTOR cascade, although the mTOR regulation of cancer cell invasion was recently shown [35, 36].

The next step of the investigation was to explore the exact mechanisms of mTOR signaling pathway in CXCR7-mediated migration and invasion of PC cells. Following use of two mTOR inhibitors, rapamycin and Torin1, in CXCR7 stably overexpressed cells, increased migration and invasion in contrast to control cells were reversed (Figure 4A). Moreover, similar phenomena were also found in expression of p-mTOR, p-4EBP1 and p-P70S6K, but not Akt and p-Akt (Figure 4B). Immunoprecipitation showed that CXCR7 directly interacts with mTOR (Figure 4C). These data confirmed that mTOR is the starting point and a key molecule of migration and invasion in PC cells, mediated by CXCR7, once again. Subsequently, the finding that Rho/ROCK pathway, that was associated with mTOR, was regulated by CXCR7 suggests that CXCL12-CXCR7-mTOR cascade might be extended as CXCL12-CXCR7-mTOR-Rho/ROCK (Figure 4D). Previously, the crucial impact of Rho/ROCK pathway on motility and migration of cancer cells, including PC ones [37, 38], was reported. Therefore, the authors give a reasonable explanation of the molecular mechanism for PC invasion induced by CXCL12/CXCR7 axis.

To verify the roles of CXCR7 in PC in vivo, we evaluated primary tumor growth and hepatic metastasis in an orthotopic implantation model in nude mice. The finding that hepatic metastatic nodule number, but not primary tumor growth, varied according to CXCR7 status is quite consistent with results derived from PC cell lines (Figure 6), and provides additional support to CXCR7 as a central modulator of invasive proclivity in PC.

Taken together, our data demonstrated that CXCL12-CXCR7 axis accelerates migration and invasion of PC cells through mTOR and Rho/ROCK pathways, and is predictive for gloomy prognosis of PC. Thus, this axis might function as a potential therapeutic target and a valuable prognostic indicator in PC.

## MATERIALS AND METHODS

### Patients

A total of 429 patients with PC who underwent surgical resection, including 235 in Beijing and 194 in Shanghai cohorts, were enrolled. There were 150 males and 85 females (median age: 61; range: 34-85 years) in Beijing cohort, and 108 males and 86 females (median age: 65; range: 38-90 years) in Shanghai one. Histological grade, T and N stage were determined by routine pathologic examinations after surgery. The clinicopathologic features of two cohorts are summarized in Supplementary Tables S1 and S2. This project was approved by Institutional Ethics Committees of the two hospitals, respectively. And, the written informed consent was obtained from the patients.

### Immunohistochemical staining, scoring and follow-up

Antibodies against human CCXL12 (R&D, Minneapolis, MN) and CXCR7 (Abcam, Cambridge, UK), and a two-step immunohistochemical staining kit (EnVision^TM^ + kit, Dako, Denmark) were used for staining. In brief, 4 μm-thick slides were mounted, deparaffinized, rehydrated. After washed with PBS, slides were autoclaved for antigen retrieval, using 0.01M citrate buffer (pH 6.0) for 10min. Slides were then incubated with 3% hydrogen peroxide for 10min to block endogenous peroxidase, followed by incubation with primary antibodies at 4°C overnight. After washing with PBS, reactions with horseradish peroxidase (HRP)-labeled secondary antibodies were performed for 30min. Diaminobenzidine was adopted as a chromogen. Finally, slides were counterstained with hematoxylin. Non-immune homologous serum at the same dilution served as the negative control.

Two pathologists who had no knowledge of the clinicopathologic and follow-up data (Z.Y. L. and W.X. Z.) independently evaluated the slides. When they were divergent, joint re-evaluations for consensus were performed. According to the criteria previously reported [27], the positive cell proportion was classified into four grades (0%, none; 1, 1-30%; 2, 31-60%; 3, >60%). And, the staining intensity was graded from 0 to 3 (0, no staining; 1, weak staining; 2, moderate staining; 3, strong staining). After multiplication of the two grades, a total staining score for one section was obtained. Final expression of CXCL12 and CXCR7 was get by a simplified classification (low expression, scores 0-3; high expression, scores 4-9).

In Beijing cohort, 182 patients (77.4%) underwent follow-up after operation, with periods ranging from 2.0 to 87.0 months (median, 12.6 months). Additionally, the follow-up periods of 153 patients (78.9%) in Shanghai cohort ranged from 2.0 to 95.0 months (median, 10.9 months).

### Cell lines and reagents

Six human pancreatic cancer cell lines, AsPC-1, BxPC-3, Mia PaCa-2, PANC-1, SU86.86 and T3M4 (kind gifts from Professor Helmut Freiss, Heidelberg University, Germany) were cultured in DMEM or RPMI 1640 media (Hyclone, Thermo Fisher Scientific Inc, Waltham, MA) supplemented with 10% fetal bovine serum (FBS, Hyclone), in a humidified incubator with 5% CO_2_ at 37°C. Extracellular matrix (ECM) and AMD3100, the specific inhibitor of CXCR4 [39], were purchased from Sigma-Aldrich (St. Louis, MO). Recombinant CXCL12, rapamycin and the antibody against CXCL12 were products of R&D Systems. Torin1 [40] and antibodies for Western blotting of CXCR7, CXCR4 and VEGF were got from Abcam. The antibody for immunoprecipitation of CXCR7 was produced by Thermo. Other antibodies for immunoblotting were all obtained from Cell Signaling Technology (Beverly, MA).

### Plasmids, siRNA and generation of CXCR7 stably silenced and overexpressed sub-lines

CXCR7 cDNA was first amplified by PCR and then sub-cloned into the pcDNA3.1 vector (Invitrogen, Carlsbad, CA). The artificial miRNA (amiRNA) duplexes (sense: 5′-TGCTGTG AAGATGAAGGCCTTCATCAGTTTTGGCCACTGACTGACTGATGAAGCTTCATCTTCA-3′; antisense: 5′-CCTGTGAAGATGAAGCTTCATCAGTCAGTCAGTGGCCAAAACTGATGAAG GCCTTCATCTTCAC-3′) were synthesized for CXCR7 silencing. Scrambled sequences were used as the controls. The duplexes were inserted to the vector pcDNA6.2 (Invitrogen) for reconstructions. The recombinant lentiviruses were packaged using the pLenti6.2 miR RNAi expression system for knockdown or the pLent6.31 expression system for overexpression (Invitrogen).

### Cell proliferation, migration and invasion assays

Cell proliferation was measured using a cell count kit (CCK-8). Following incubation with cell culture media containing CCK-8 reagent for 3h, absorbance at 450nm was detected by a microplate enzyme-linked immunosorbent assay reader (Wellscan MK3, Thermo/Labsystems, Finland).

Transwell inserts (pore size: 8.0μm, Corning, Chelmsford, ST) were used in migration and invasion assays. For cell migration, 500μL of medium with 10% FBS was filled to the lower chamber. Cells (BxPC-3: 3×10^5^; AsPC-1: 4×10^5^) were re-suspended in serum-free media and added to the upper chamber. After an incubation of 24h at 37°C, cells on the upper surface of the membrane were carefully scraped out. Migrated cells that were adherent to the lower surface of the membrane were fixed in 10% formalin. Then, hematoxylin and eosin staining was performed. Finally, the cells were counted in five fields at a magnification of ×200.

For cell invasion, the under surface of the membrane was coated with ECM gel (Sigma-Aldrich). A total of 6×10^5^ (BxPC-3) and 8×10^5^ (AsPC-1) cells were used. The next steps same with those of migration assay were finished one by one. Each experiment was repeated for three times.

### Western blotting and immunoprecipitation

Extracted total proteins (80μg/lane) were separated by sodium dodecyl sulfate polyacrylamide gel electrophoresis (SDS-PAGE) and transferred to a polyvinylidene difluoride (PVDF) membrane (Millipore, Billerica, MA). Following blocked with 5% non-fat dry milk, the membrane was incubated overnight at 4°C with primary antibodies. Horseradish peroxidase-conjugated secondary antibodies were then added for reactions at room temperature. Protein bands were visualized by enhanced chemiluminescence reagents (Merck, Darmstadt, Germany). Beta-actin was applied as the internal control. For immunoprecipitation, cell lysates were incubated with appropriate primary antibodies for 12h at 4°C, followed by addition of protein A agarose beads. The immunoblotting with secondary antibodies was then carried out. All experiments were performed in triplicate.

### Xenograft experiments

A total of 64 female BALB/c nude mice that were six weeks old were obtained from the Chinese Academy of Medical Sciences (CAMS), Beijing, China, and were maintained under specific pathogen-free conditions. The mice were randomly divided into eight groups, according to different cell types. Cells (2×10^6^/20μL) were injected into the pancreas of each nude mouse during open laparotomy. After 6 weeks, the mice were sacrificed. Orthotopic pancreatic tumors were measured with calipers in two dimensions, their volumes were calculated. Besides, the numbers of hepatic metastatic lesions were counted in consecutive slides made for each tissue block after hematoxylin and eosin (H&E) staining. All of the experiments were approved by the Animal Care and Use Committee of CAMS.

### Statistical analysis

Comparisons of continuous variables were performed by Student *t*-test. CXCL12 and CXCR7 staining scores between tumor and non-tumor tissues were compared using Mann-Whitney *U* test. Chi-square test was applied to show associations between staining scores and clinicopathologic parameters. Overall survival was calculated by Kaplan-Meier method and analyzed by log-rank test. Cox regression (Proportional hazard model) was employed for multivariate analysis of prognostic factors. All the analyses were performed using Statistical software package SPSS11.5 (SPSS Inc, Chicago, IL). A statistically significant *P* value was defined as <0.05.

## SUPPLEMENTARY FIGURES AND TABLES


