# Supplementary material for: CXCL12-CXCR7 axis contributes to the invasive phenotype of pancreatic cancer
Source: Oncotarget. 2016 Aug 17;7(38):62006–18. doi: 10.18632/oncotarget.11330 (PMC5308707; doi:10.18632/oncotarget.11330)
Supplement: Supplementary file 1 [file oncotarget-07-62006-s001.pdf]

CXCL12-CXCR7 axis contributes to the invasive phenotype of pancreatic cancer

SUPPLEMENTARY FIGURES AND TABLES

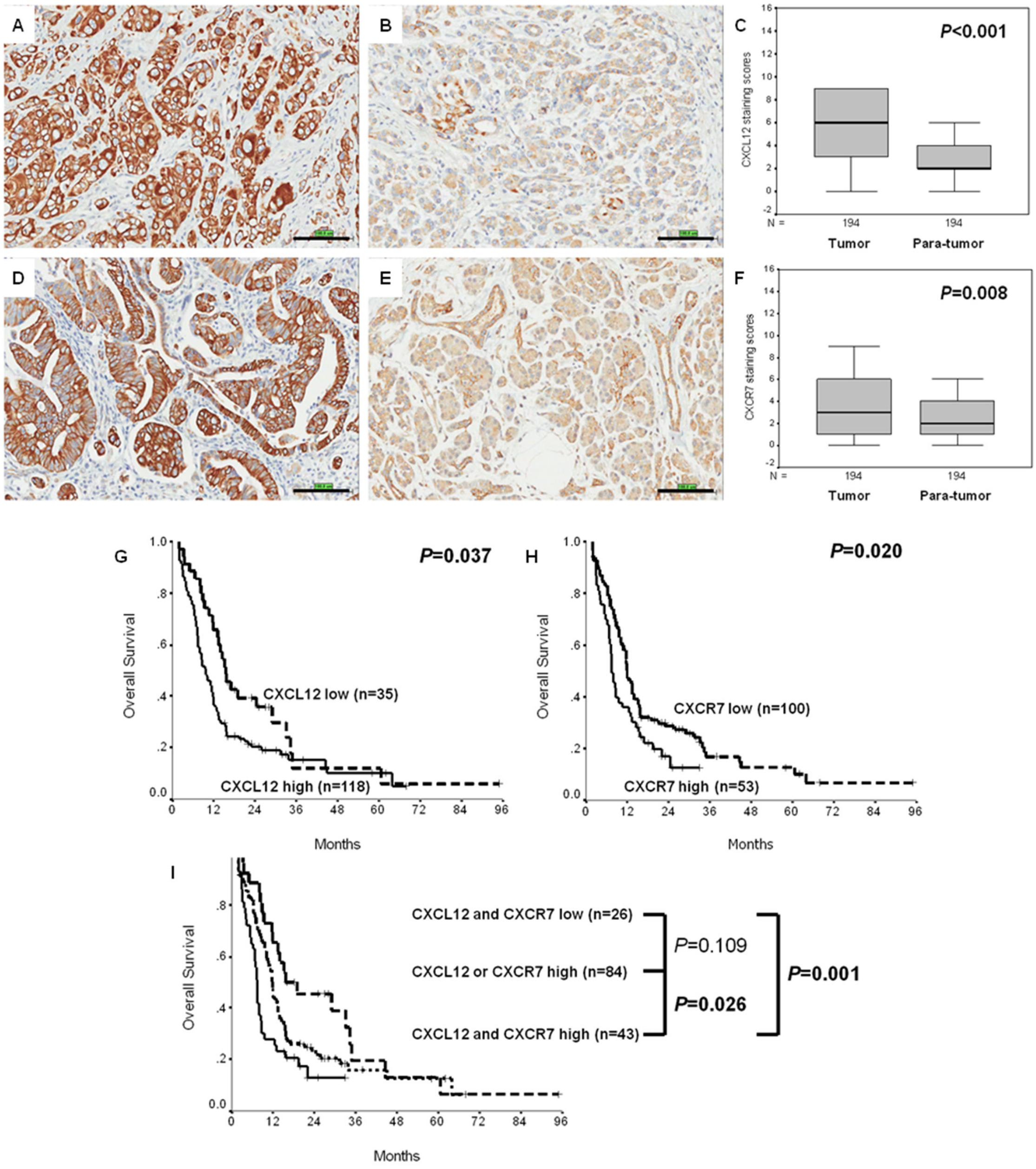

Supplementary Figure S1: Expression, clinicopathologic and prognostic significances of CXCL12 and CXCR7 in Shanghai cohort of PC. A. High CXCL12 expression in tumor tissues (×200). **B.** High CXCL12 expression in non-tumor tissues (×200). **C.** Staining ranks of CXCL12 in tumor tissues were statistically higher than those in non-tumor ones. **D.** High CXCR7 expression in tumor tissues (×200). **E.** High CXCR7 expression in non-tumor tissues (×200). **F.** Staining ranks of CXCR7 in tumor tissues were statistically higher than those in non-tumor ones. **G.** High CXCL12 expression in tumor tissues predicted poor overall survival. **H.** High CXCR7 expression in tumor tissues predicted poor overall survival. **I.** Coexpression of CXCL12 and CXCR7 carried poorest overall survival (The upper line: CXCL12 and CXCR7 low; the medial line: CXCL12 or CXCR7 high; the nether line: CXCL12 and CXCR7 high).

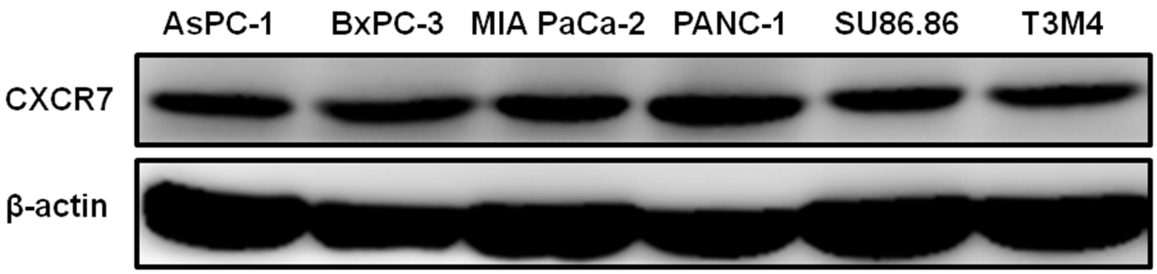

Supplementary Figure S2: Baseline expression of CXCR7 in six PC cell lines.

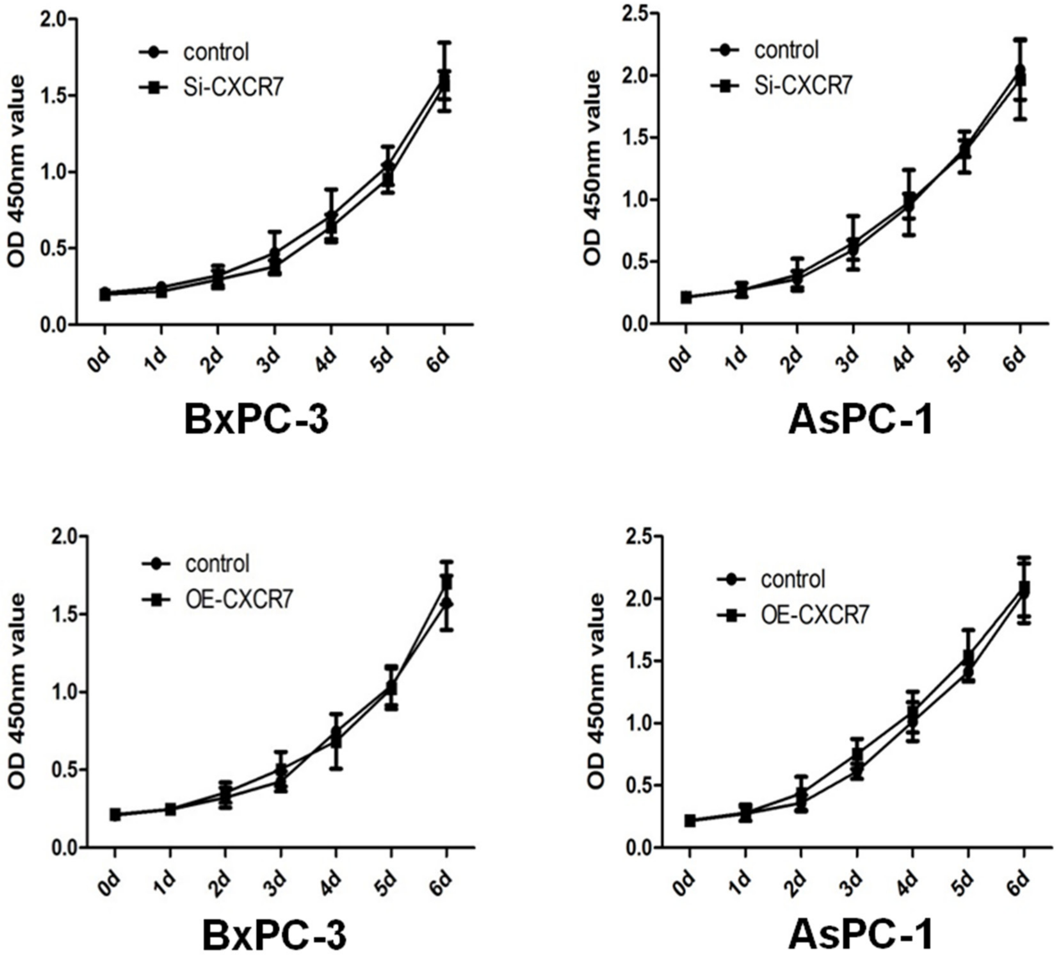

Supplementary Figure S3: Stable silence and overexpression of CXCR7 do not affect proliferation in PC cells.

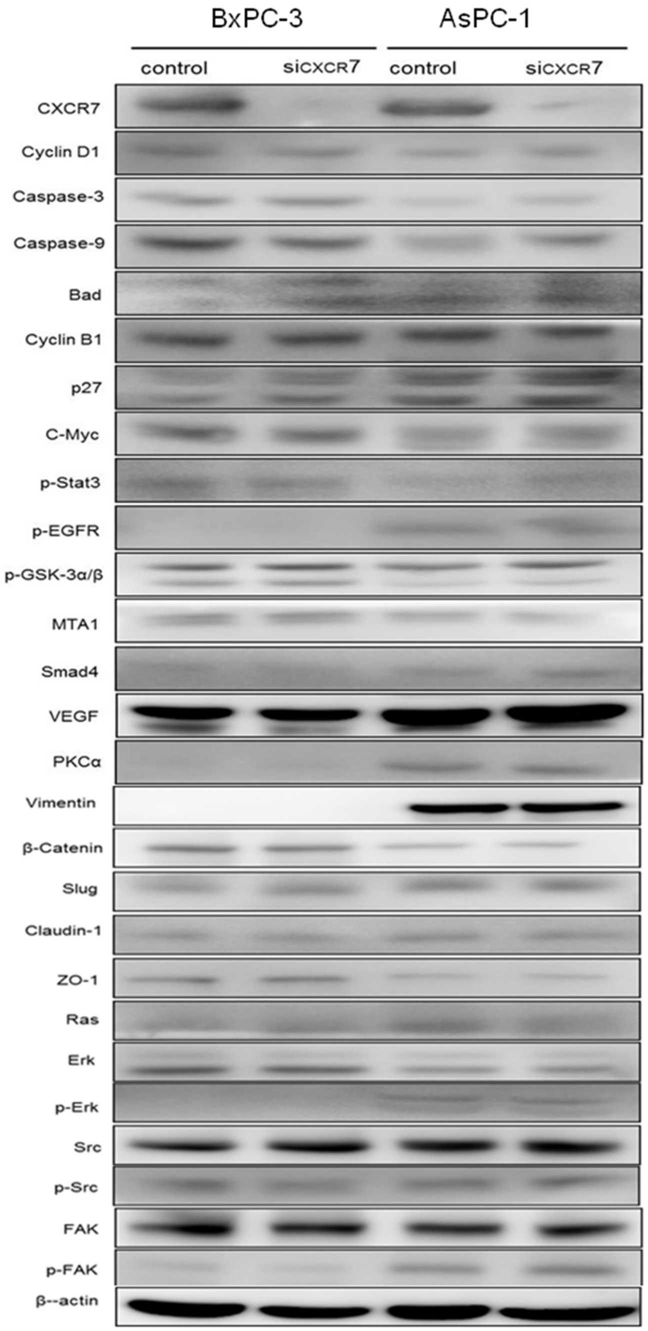

Supplementary Figure S4: Stable silence of CXCR7 does not influence expression and phosphorylation of a panel of proteins in PC cells.

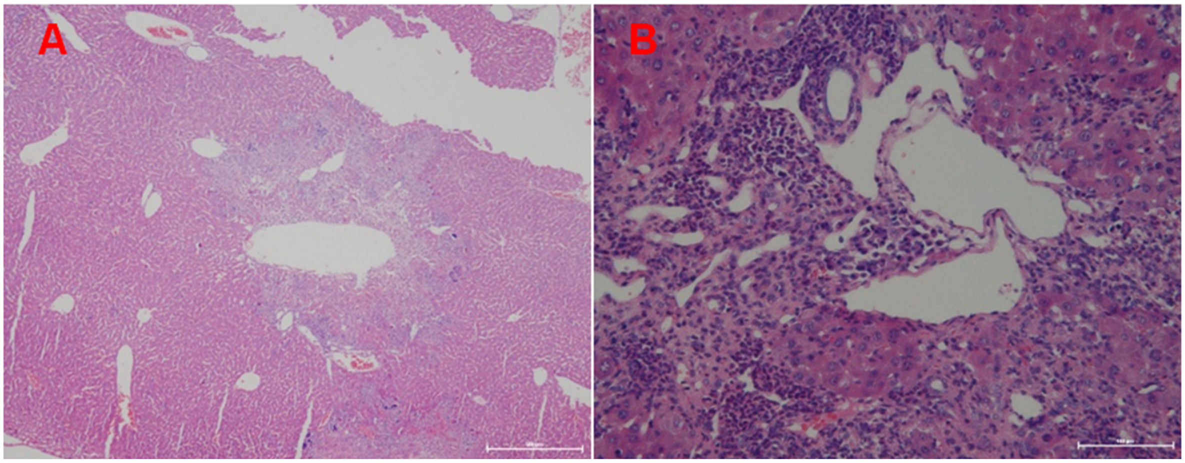

Supplementary Figure S5: Hepatic metastasis of PC in nude mice. A. Liver tissue with a metastatic lesion (×40). **B.** Metastatic lesion (×200).

Supplementary Table S1: CXCL12/CXCR7 expression and clinicopathologic variables of PC (Beijing cohort)

Variables	n	CXCL12 expression		*P*	CXCR7 expression		*P*
		High	Low		High	Low	
Age				0.276			0.738
≥65 years	77	51	26		30	47	
<65 years	158	93	65		58	100	
Sex				0.255			0.147
Male	150	96	54		51	99	
Female	85	48	37		37	48	
Tumor location				0.216			0.659
Head	140	83	57		51	89	
Non-head	89	60	29		35	54	
Tumor size				0.968			0.403
>4cm	89	55	34		30	59	
≤4cm	143	88	55		56	87	
Histological grade				**0.019**			0.392
G1-2	154	87	67		60	94	
G3-4	64	47	17		21	43	
Vessel invasion				**0.007**			**0.022**
Present	102	73	29		46	56	
Absent	131	71	60		40	91	
T stage				0.116			0.081
T1-2	141	92	49		46	95	
T3	91	50	41		40	51	
N stage				0.583			0.939
N0	117	68	49		43	74	
N1	102	63	39		38	64	

NOTE: Partial data are not available, and statistics were based on available data. *P* values were derived from the Pearson Chi-square test (two-tailed).

Supplementary Table S2: CXCL12/CXCR7 expression and clinicopathologic variables of PC (Shanghai cohort)

Variables	n	CXCL12 expression			CXCR7 expression		
		High	Low	*P*	High	Low	*P*
Age				0.125			0.558
≥65 years	105	79	26		36	69	
<65 years	89	58	31		27	62	
Sex				0.932			**0.033**
Male	108	76	32		42	66	
Female	86	61	25		21	65	
Tumor location				0.545			0.364
Head	130	90	40		45	85	
Non-head	64	47	17		18	46	
Tumor size				0.995			0.190
>4cm	68	48	20		18	50	
≤4cm	126	89	37		45	81	
Histological grade				0.400			**0.045**
G1-2	124	85	39		34	90	
G3-4	70	52	18		29	41	
Vessel invasion				**0.047**			**0.003**
Present	33	28	5		18	15	
Absent	160	108	52		45	115	
T stage				0.529			0.272
T1-2	26	17	9		6	20	
T3	168	120	48		57	111	
N stage				0.409			0.486
N0	132	90	42		41	91	
N1	58	43	15		21	37	

NOTE: Partial data are not available, and statistics were based on available data. *P* values were derived from the Pearson Chi-square test (two-tailed).

Supplementary Table S3: Univariate and multivariate analyses of prognostic factors of PC (Shanghai cohort)

Variables	n	Univariate			Multivariatet		
		median±SE	95% CI	*P*	HR	95% CI	*P*
Age				0.075			
≥65 years	90	8.7±2.4	6.0-11.4				
<65 years	63	13.2±1.2	10.8-15.6				
Sex				0.263			
Male	84	10.9±1.0	8.9-12.9				
Female	69	11.5±1.9	7.8-15.2				
Tumor location				0.715			
Head	102	11.5±1.0	9.6-13.4				
Non-head	51	10.3±1.3	7.7-12.9				
Tumor size				0.180			
>4cm	50	8.3±1.4	5.5-11.1				
≤4cm	103	12.0±0.9	10.3-13.7				
Histological grade				**0.001**			**0.035**
G1-2	93	12.4±1.2	10.0-14.8		1		
G3-4	60	8.0±0.7	6.6-9.4		1.527	1.030-2.263	
Vessel invasion				**0.017**			
Present	26	6.8±1.1	4.7-8.9				
Absent	126	11.7±0.8	10.0-13.4				
T stage				0.325			
T1-2	19	14.0±2.7	8.7-19.3				
T3	134	10.3±1.0	8.4-12.2				
N stage				**0.033**			**0.037**
N0	100	12.0±1.0	10.0-14.0		1		
N1	50	8.5±1.2	6.1-10.9		1.508	1.025-2.220	
Tumoral CXCL12				**0.037**			0.054
High	118	9.6±1.1	7.5-11.7		1.513	0.992-2.305	
Low	35	15.4±1.7	12.1-18.7		1		
Tumoral CXCR7				**0.020**			0.062
High	53	7.5±0.6	6.4-8.6		1.451	0.982-2.144	
Low	100	11.9±0.9	10.2-13.6		1		

NOTE: Partial data are not available, and statistics were based on available data. *P* values were derived from the Log-rank test (univariate) and Cox regression analysis (multivariate).
